# Preventive measures for development of contrastassociated acute kidney injury in critical patients. Preliminary results of the nefrocon study

**DOI:** 10.1186/2197-425X-3-S1-A52

**Published:** 2015-10-01

**Authors:** S Mas-Font, C Gómez-Gonzalez, M Herrera-Gutierrez, FJ Gonzalez de Molina, R Catalán-Ibars, S Aldunate-Calvo

**Affiliations:** Hospital Universitario General de Castellón, Intensive Care Unit, Castellón, Spain; Hospital Infanta Luisa, Intensive Care Unit, Sevilla, Spain; Complejo Universitario Carlos Haya, Intensive Care Unit, Málaga, Spain; Hospital Universitari Mutua de Terrassa, Terrassa, Spain; Hospital General de Vic, Intensive Care Unit, Barcelona, Spain; Complejo Hospitalario de Navarra, Intensive Care Unit, Pamplona, Spain

## Objectives

The aim of this study is to analyze the effect of preventive measures for development of contrast associated acute kidney injury (CA-AKI), controlling the maximum number of factors confounders using propensity score methodology.

## Methods

We performed a prospective multi-center study, in 34 Spanish ICU, covering the period from 15 December 2012 to 15 March 2013. During this study period, we included 1035 patients, all of them undergoing a radiographic examination or a coronary angiography with administration of parenteral iodinated contrast media. We excluded patients with incomplete data or renal replacement therapy at the time of the study, being finally 1012 patientes. We defined CA-AKI as an increase of serum creatinine ≥, 0,5 mg/dl, or ≥ 50 % from baseline, assessed 48-72 hours after the procedure. We calculate a propensity score through regression and subsequent pairing 1:1 by quintiles clustering. The final analysis was performed using logistic regression, including the propensity score and factors that could not be balanced.

## Results

Preventive measures were applied in 29,4% of patients: 53,8 % surgical patients, 30,1% medical patients, and 18,6 % coronary patients, p < 0,001. The most common choice was volume loading (22,6%), especially in younger patients (60.02-0.94 vs 63.02-0.55, p < 0.05), more severely ill patients (APACHE 14,3-0,45 vs 11.9-0.28, p < 0.001), and patients with worse baseline creatinine (1.08-0.35 vs 0.94-0.01, p < 0.001) and at time of contrast administration (1.24-0.46 vs 1.01-0.02, p < 0.001). Also we applied more preventive measures in patients with chronic kidney disease stage ≥ 2. The incidence of CA-AKI was higher in patients who received preventive measures, OR 1.73, (95% CI 1.17-2.56) and an absolute difference of 6,2% (IC 1,2-11,2). We estimate a propensity score considering: type of hospital and patient, age, chronic kidney disease, baseline serum creatinine, APACHE II, serum creatinine at time of contrast administration, volume of contrast, glucose and hemoglobin at time of contrast administration, vasoactive therapy, NSAID, ACEI and shock. In the final regression analysis, we included the type of patient, volume of contrast, use of ACEIS and glucose and hemoglobin levels. After pairing, OR for preventive measures was 1,19 (0,71-2,14), and finally, after controlling for other variables mentioned, we obtained an increase in the risk of CA-AKI in patients with prophylaxis of 1,1 (0,7-1,72), no significant.

## Conclusions

CA-AKI is apparently more frequent after the application of preventive measures, this is due to the numerous variables which interfere in the decision on your application. However, in our cohort we have failed to either show a definitive positive effect on its appearance.

